# Rapid Detection and Identification of Antimicrobial Peptide Fingerprints of Nasal Fluid by Mesoporous Silica Particles and MALDI-TOF/TOF Mass Spectrometry: From the Analytical Approach to the Diagnostic Applicability in Precision Medicine

**DOI:** 10.3390/ijms19124005

**Published:** 2018-12-12

**Authors:** Mariaimmacolata Preianò, Giuseppina Maggisano, Maria Stella Murfuni, Chiara Villella, Carmela Colica, Annalisa Fregola, Corrado Pelaia, Nicola Lombardo, Girolamo Pelaia, Rocco Savino, Rosa Terracciano

**Affiliations:** 1 Department of Health Sciences, Laboratory of Mass Spectrometry and Proteomics, “Magna Græcia” University, 88100 Catanzaro, Italy; preiano@unicz.it (M.P.); giusimaggisano@unicz.it (G.M.); murfuni@unicz.it (M.S.M.); villella@unicz.it (C.V.); annalisafregola@gmail.com (A.F.); savino@unicz.it (R.S.); 2 CNR, IBFM UOS of Germaneto, “Magna Græcia” University, 88100 Catanzaro, Italy; carmela.colica@cnr.it; 3 Department of Medical and Surgical Sciences, “Magna Græcia” University, 88100 Catanzaro, Italy; pelaia.corrado@gmail.com (C.P.); nlombardo@unicz.it (N.L.); pelaia@unicz.it (G.P.)

**Keywords:** antimicrobial peptides, peptidomics, MALDI-TOF MS, mass spectrometry, nasal fluid, biomarkers, nasal polyposis, precision medicine, biomarker, enrichment

## Abstract

Background: Antimicrobial peptides (AMP) play a pivotal role in innate host defense and in immune response. The delineation of new MS-based profiling tools, which are able to produce panels of AMP of the nasal fluid (NF), may be attractive for the discovery of new potential diagnostic markers of respiratory disorders. Methods: Swabs collected NF from healthy patients and from patients with respiratory disorders. We used a fast procedure based on mesoporous silica particles (MPS) to enrich NF in its AMP component in combination with MALDI-TOF/TOF MS as a key tool for rapidly analyzing clinical samples. Results: Reproducible MS peptide fingerprints were generated for each subject and several AMP were detected including (Human Neutrophil Peptides) HNPs, Statherin, Thymosin-β4, Peptide P-D, II-2, β-MSP, SLPI, Lysozyme-C, and their proteo-forms. In particular, Statherin, Thymosin-β4, and Peptide P-D were accurately identified by direct MS/MS sequencing. Examples of applicability of this tool are shown. AMP fingerprints were obtained before and after a nasal polypectomy as well as before and post-treatment with azelastine/fluticasone in one case of allergic rhinitis. Conclusion: The potential of our platform to be implemented by new mesoporous materials for capturing a wider picture of AMP might offer an amazing opportunity for diagnostic clinical studies on individual and population scales.

## 1. Introduction

The nose is the part of the respiratory system in which it is easy to study morphological and pathophysiological changes due to inflammatory responses to various stimuli because of its accessibility [1]. The respiratory tract is constantly challenged with airborne and aspirated microbes and, as part of the nose’s functions, the nasal epithelium along with a mucous airway surface liquid, which provides continuous removal of inhaled microbes targeting lower airways [2]. In particular, important defense functions are due to the presence in nasal secretions of AMP such as lactoferrin, defensins, lysozyme, peptide LL37, secretory leukocyte protease inhibitor (SLPI), β-microseminoprotein (β-MSP), statherin, and other antimicrobial components [3]. The majority of AMP are cationic and amphipathic. However, there are also hydrophobic α-helical peptides with antimicrobial activity. Most AMP interact with membranes and may be cytotoxic since they disrupt the functions of the bacterial inner or outer membranes. Moreover, their functions in the innate host defense is also to modulate the immune response through both chemotaxis and neutralization of proinflammatory microbial products [4]. Some AMP play an important role by alerting the adaptive immune system of the presence of infections agents. Several pathologies characterized by persistent mucosal colonization or infection, among which cystic fibrosis and nasal colonization with *Staphylococcus aureus*, may be due to impaired AMP-based defenses [5]. Nasal fluid (NF) is an important diagnostic source of biomarkers for a large plethora of disorders of the respiratory tract. Specifically, the analysis of AMP as immunological markers in nasal secretions provides valuable information for monitoring immunotherapy and the immune response to vaccines administered locally [6]. Additionally, given the well-established similarities in mucosal activity between upper and lower airways, the study of the expression of AMP in the nasal cavity may be useful for a better understanding of the mechanism of several airway diseases including those of the lower tract with the benefits of an easy and non-invasive sample collection and a consequent better patient compliance. Within this context, proteomic and peptidomic techniques are emerging as highly valuable tools that can be applied to the analysis of nasal mucosa secretions. Standardized and reproducible samples of both nasal exudates and mucosal cells can be obtained in a relatively noninvasive manner such as nasal lavage, nasal swabs, filter paper strips, nasal brushing and scraping, and a nasal biopsy [1,7]. These specimens constitute a useful source of information for clinical-based and large-scale proteomics investigations. Up to date, however, the nasal cavity and mucous proteome have been explored only by a small cohort of studies [8,9,10,11,12]. Specifically, a limited number of MS–based proteomic tools were used for the comparative analysis of peptidome and proteome changes in rhino-pathology and respiratory disorders such as allergic and non-allergic rhinitis [7,8,13,14,15,16,17,18], respiratory infections and cystic fibrosis [19,20,21], chronic sinusitis, and rhinosinusitis [13,22,23].

As a part of an ongoing project aimed for the rapid MS-profiling of endogenous peptides from bodily fluid to be used in comparative clinical studies, we standardized a standard procedure for the collection of NF by the nasal swab procedure for proteomic/peptidomic profiling. Based on the combined use of MPS and MALDI-TOF MS, our rhinomics platform was able to generate highly reproducible peptide profiles [7]. In the present investigation, we carried out the identification and characterization of further endogenous peptides of NF selectively adsorbed by MPS, which revealed the presence of important AMP. The delineation of new platforms, which are able to produce panels of AMP present in the NF, may be of interest not only for the discovery of new potential diagnostic markers of the respiratory tract but also to reveal the occurrence of post-translational modifications, polymorphisms, and other proteoforms, which could be relevant in the innate host defense. In the present investigation study, we describe and highlight the usefulness of our novel MPS-MALDI-TOF/TOF “rhinomics” platform for the rapid visualization and characterization of the AMP fingerprint of NF, which is described and highlighted. This envisions as proof-of-concept its potential applicability in a clinical and diagnostic setting and opens new perspectives for personalized management of respiratory diseases. 

## 2. Results and Discussion

**Figure 1:**
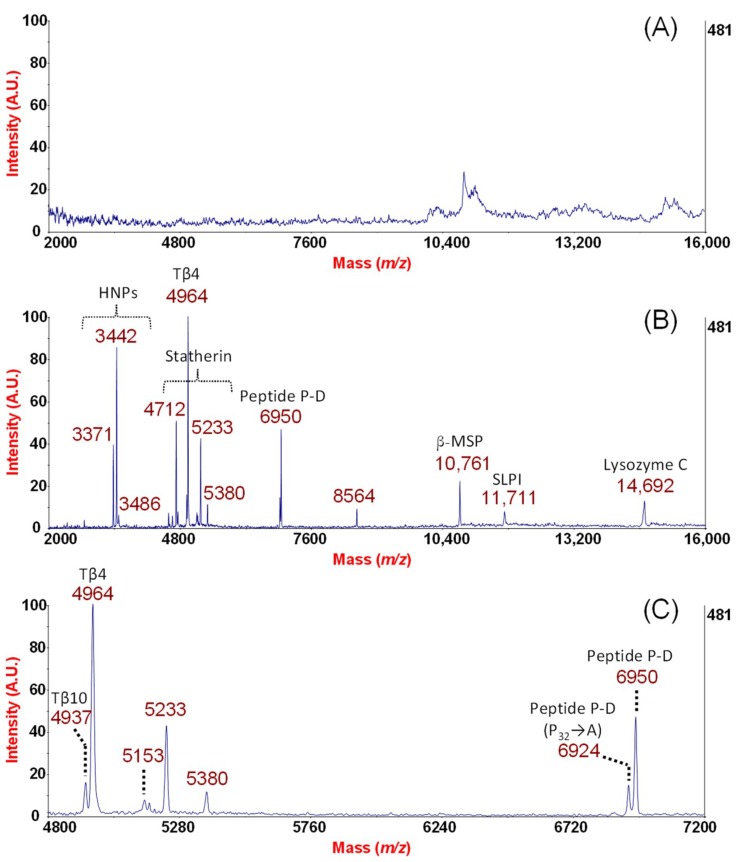
MALDI-TOF mass spectra of NF before (**A**) and after processing by MPS-C (**B**). A nasal swab collected nasal fluid (NF) from one healthy participant in the study and 30% of sodium chloride was used as eluent (**A**). The eluate was then incubated with the MPS particles for the selective enrichment of nasal antimicrobial peptidome. MALDI measurements were performed in sinapinic acid (SA) and the spectra were recorded in linear mode in the *m*/*z* range 2000–20,000. Molecular mass windows are shown in the *m*/*z* range between 2000 and 16,000 and (**B**) between 4800 and 7200 (**C**).

High molecular weight glycoproteins such as mucins may constitute an obstacle for the MS detection of peptides in nasal secretions. We have recently described a rapid procedure for displaying endogenous peptides in the NF of healthy, allergic, and non-allergic rhinitis subjects based on the combined use of MPS and MALDI-TOF MS profiling [7]. Based on a cut-off mechanism and on a result of a variety of interactions, MPS capture small-sized molecules for instance endogenous peptides of NF that are able to diffuse into the nanometric pore diameter of the meso-pores. Therefore, larger-sized proteins (like mucins and polymucosaccharides) that compete with the desorption/ionization process and could mask and/or suppress peptidome signals in MALDI-TOF analysis are excluded from this adsorptive process [7]. In particular, continuing our previous investigations in this study, we observed that aminopropyl-functionalized MPS-C, among other kind of MPS, are able to enrich NF in its antimicrobial peptidic components in a molecular weight range from 3000 to 15,000 Da. Figure 1 shows the MALDI-TOF profiles of one out of the eight healthy subjects examined. In particular, as shown in Figure 1A, without any processing with MPS, the MALDI-TOF spectrum of NF displays only some sporadic or rare peaks. The use of MPS-C appears to provide sufficiently rich fingerprints of NF, which was revealed by comparing the mass spectra of NF eluted from the swab before (Figure 1A) and after (Figure 1B) the extraction procedure. In particular, Figure 1B shows the peptide repertoire of NF selectively adsorbed into the mesoporous channels of the MPS-C. MPS with their great surface area provide a high binding capacity and, moreover, the possibility of thoroughly washing the particles allows the effective removal of salts and other contaminants, which resulted in high quality spectra. The use of porous particles for sample pre-treatment is more sensitive than the surface capture on chips (characteristic of SELDI-TOF technology) because MPS have larger combined surface areas than the SELDI flat chips [24]. We tried to assign all these peaks by MALDI-TOF/TOF MS for sequence identification directly from NF processed with the MPS or alternatively after digestion of a selected band from 1D SDS-PAGE of NF proteins. The triplet in the range between 3 and 4 kDa matched with the HNPs HNP-1 (*m*/*z* = 3442), HNP-2 (*m*/*z* = 3371), and HNP-3 (*m*/*z* = 3486), which are also known as human α-defensins (Figure 1B). In the range between 4.5 and 5.5 kDa, we identified Statherin (*m*/*z*= 5380), its main fragments (*m*/*z* = 4712 and *m*/*z* = 5233), and Thymosin-β4 (*m*/*z* = 4964) (Figure 1B). Two basic proline rich peptides were also detected: Peptide P-D (*m*/*z* = 6950) (Figure 1B) and phosphorylated II-2 (*m*/*z* = 7608) (Figure S1). The main representative features in the region from 10,000 to 16,000 are the peaks at *m*/*z* = 10761, *m*/*z* = 11711, and *m*/*z* = 14692, which were identified as β-MSP, SLPI, and Lysozyme C, respectively (Figure 1B). Our platform allows the detection of polymorphism for an endogenous peptide. For instance, peptide P-D and, in such a case, it was possible to detect Thymosin-β10 as a homologue form of Thymosin-β4 (Figure 1C). The peak doublet characteristic for the isoforms of peptide P-D is well highlighted in Figure 1C in which the variant due to one aminoacid replacement P32A (*m*/*z* = 6924) is shown together with Thymosin-β4 homologue, Thymosin-β10 (*m*/*z* = 4937). 

**Figure 2:**
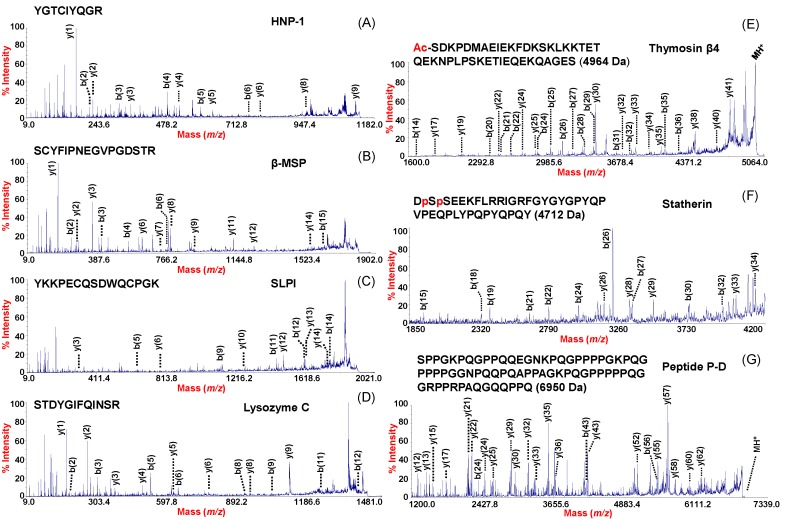
MALDI-TOF/TOF mass spectra of some relevant peptides analyzed by sequence identification directly from NF processed with the MPS or alternatively after gel electrophoresis separation of the captured proteins. The left column shows MS/MS spectra of peptides obtained from triptic digestion of (**A**) HNP1: YGTCIYQGR (residues 80 to 88). (**B**) β-MSP: SCYFIPNEGVPGDSTR (residues 21 to 36). (**C**) SLPI: YKKPECQSDWQCPGK (residues 46 to 60) and (**D**) Lysozyme C: STDYGIFQINSR (residues 69 to 80). The right column shows MS/MS spectra obtained by direct sequencing of (**E**) Thymosin β4 (Red letter Ac: N-terminal acetylated), (**F**) statherin Des^39–43^ (Red letter p: phosphorylated on Ser 2 and Ser 3), and (**G**) Peptide P-D.

Compared to the chip-to-chip variability and low resolution SELDI MS data, besides lower analytical variability and higher resolution, another advantage of our MPS-MALDI-TOF/TOF platform is given by its flexible workflow with an accurate high resolution identification of peptides. Given the performances of the MALDI coupled to a double TOF analyzer, it is also possible to identify specific peptides detected in the mass spectra by direct sequencing. The peaks of interest were analyzed by MALDI-TOF/TOF MS for sequence identification directly from NF processed with the MPS or alternatively (when our attempts to sequence directly by MALDI-TOF/TOF resulted in a low quality MS/MS spectra with low-confidence identification) by peptide mass fingerprinting after gel electrophoresis separation and tryptic digestion of the selected band. The identified peptides are listed in Table 1. The most representative mass spectra with the mass sequence assignment of the identified peptides are shown in Figure 2. The main classes of NF peptides detected and identified in the present study are the α-defensins (3371 Da, 3442 Da, 3486 Da), which are also known as HNPs (HNP1-3), the β4-Thymosin (4964 Da), the peptide P-D (6950 Da), the β-MSP (10,761 Da), SPLI (11,711 Da), Statherin and its fragments (5380 Da, 5233 Da and 4712 Da), and Lysozyme C (14,692 Da).

Many of these AMP detected are well-known components of the antimicrobial rhino-mucosal barrier. Specifically, β-MSP, SLPI, and Lysozyme were previously detected and identified in nasal secretions [3]. NanoHPLC-MALDI-TOF/TOF MS identified shorter Peptide P-D and Sthaterin derived fragments detected in saliva degradome, which were identified by nanoHPLC-MALDI-TOF/TOF MS [25]. The peptides shown in Table 1 play an essential role in innate immunity, are mainly produced by epithelial cells and neutrophils, and kill a wide range of bacteria, fungi, and viruses [26]. We are conscious that MPS-C does not allow the detection of other AMP such as secretory Phospholipase A2 (sPLA2), Human β-defensin 1 (HBD-1) and Human β-defensin 2 (HBD-2). However, the possibility to prepare and test novel hybrid mesoporous material in order to finely modulate the intrinsic selectivity toward cationic and/or hydrophobic peptides might offer new opportunities and challenges for our platform for capturing a wider picture of poorly expressed AMP.

### From the Analytical Approach to Diagnostic Application

According to our previous study, the enrichment strategy of NF peptides by MPS was carefully controlled. In particular, the concentrations of the solutions eluted from nasal swabs from each donor patient were normalized to obtain the same amount of unfractionated protein for further processing with MPS. In early experiments, we observed that peptides adsorption into MPS change with pH [27]. In a neutral solution, MPS-C with primary amine groups (pKa~10) retain a positively charged surface. However, different adsorption pH values were also tested in order to ensure that the maximum number of peptides were selectively captured by MPS. We found that the best working conditions (in terms of MALDI-TOF peak number detection) were obtained at pH = 8.8. In this condition, it is expected that roughly 10% of the functional groups present on MPS-C are uncharged. This furnishes an elegant explanation for the empirical observation of the high pI variability of the peptides reported in Table 1. In these conditions, it is rather straightforward to hypothesize that peptides expected to have a net negative charge (for instance Peptide P-D, pI 11.17) were captured mainly by electrostatic interactions. On the other hand, it can be speculated that peptides at a pH of 8.8 are expected to be either neutral (for instance HNPs pI 8.67) or positively charged (for instance, Thymosin β4 pI 5.02) were captured through other kinds of interactions by uncharged functional groups expected to be present on the silica surface.

Reproducibility was assessed by measuring the coefficient of variations (CVs) for a normalized area along with height and signal-to-noise ratio (S/N) of selected peaks in acquired MALDI-TOF MS spectra from independent intra-day replicate analyses (*n* = 3) of the same NF sample processed by MPS. Table S1 lists CVs of AMP selected in this study for normalized peak area, peak intensities, and S/N. The CVs for the peaks of interest (Table S1) were comprised in a range from 5% to 16%, 8% to 15%, and 8% to 17%, for normalized area, height and S/N, respectively, which confirms the robustness of the method. Analytical and instrumental conditions already tested in our previous study were carefully controlled in order to ensure accurate and reproducible measurements. We also evaluated the subject-to-subject variability in the group of the eight healthy donors. The peptide profiles shared most of the peaks detected. The peak frequency for the detected peptides is reported in Table 1. Thymosin β4, SLPI, Lysozyme-C, Peptide P-D and the Statherin fragment (*m*/*z* = 4712) were present in all donors. β-MSP and the phosphorylated form of II-2 peptide were shared by seven of the eight donors and a lower frequency was observed for the other peptides such as the alpha defensins. All subject profiles are reported in Figure S1, which clearly shows the inherent biological variability among subjects. In the case of alpha defensins, the observed amount of between-individual variability could be attributed to genetic factors affecting the phenotype (gene DEFA3/1). In particular, these HNPs were observed in five of the eight donors and, specifically, HNP_3_ was detected in only three of the five donors with HNPs (see Table 1, Figure S1). Defensin alpha 3 differs from defensin alpha 1 by only one amino acid. The genes encoding for defensin alpha 1 and defensin alpha 3 are both subject to the copy number variation. It is important to observe that this copy number polymorphism of *DEFA1*/*DEFA3* with a consequent frequent absence (7/27 individuals as reported in literature) of the *DEFA3* allele encoding the HNP-3 peptide are believed to likely affect the function and effectiveness of innate immunity [28].

Mass spectra of five donors showed doublet signals as observed before in Figure 1C for the peaks *m*/*z* = 6924 and *m*/*z* = 6950 with a difference of + 26 amu explained by an amino acid substitution in peptide P-D *m*/*z* = 6924 (P32A). The two species of P-D peptide, differing for the single substitution P32A, may be derived from all three alleles on the PRB4 locus and are detectable in parotid and whole saliva [29]. Peptide P-D (alternative name IB-5) belongs to the basic PRPs which are found in saliva. However, they are also found in bronchial and nasal secretions [30]. Interestingly, another basic PRP, II-2 was also found in the NF of the analyzed subjects, prevalently in phosphorylated form (*m*/*z* 7608) in seven out of the eight individuals, while the non-phosphorylated form (*m*/*z* 7528) was detected (mainly with a lower S/N) in five subjects (Table 1 and Figure S1). The complete understanding of all the functions of bPRPs is still missing. However, it has been shown that salivary II-2 peptide is involved in the PROP bitter taste responsiveness [31] while the salivary peptide P-D [32] is involved in tannins precipitation. We can only argue that the cationic charge in their structures might have a strong correlation with antimicrobial activity.

Inter-subject variability, assessed on the main peaks selectively adsorbed in MPS-C and detected by MALDI-TOF MS, was reported in Figure S2 where the normalized peak area are plotted with their standard deviation (SD) for the peaks of interest. A larger relative variability was mainly observed for the peaks with lower frequency (HNP-3, Thymosin β-10, unphosphorylated II-2). All these findings highlight the ability of our tool to reveal proteoforms present in the nasal cavity (including those due to polymorphism) by furnishing unique panels with characteristic AMP fingerprints influenced by both genetic and external factors. As it happens in the oral cavity for saliva [33] and gingival crevicular fluid [34], we do not exclude that the observed inter-individual variability might also be due to the action of endo-proteases including those of microbial origin in the nasal environment, which give rise to further fragmentation of naturally occurring polypeptides. Therefore, this platform might be useful not only to study the frequency of such proteoforms but also to study, on a larger cohort, the influence on the AMP constituents in NF of genetic and external factors including the environment, exposition, gender, and age in order to better understand how the upper airways establish their defense functions. 

It is worth noting that, by this procedure, it is possible to convert nasal swabs into snapshots illustrating the individual peptide fingerprint with information about the quali/quantitative composition of the endogenous peptides at the time of the collection (especially the AMP present in the NF). Specifically, unique profiles of AMP can be found in nasal secretions, which could provide an appropriate response to the microorganisms invading this specific epithelium. For this reason, from a clinical point of view, this technology allows monitoring the variation of a specific antimicrobial peptidome profile at any time such as during and after a patient’s therapy or before and after surgery. To explore the possibility of using our approach as a tool for monitoring any variation within a patient in a clinical setting, we analyzed the variation of the AMP fingerprint in the case of a subject suffering of nasal polyposis (NP) before and after polipectomy and in the case of a subject suffering from allergic rhinitis (AR) before and after azelastine/fluticasone topical treatment.

**Figure 3:**
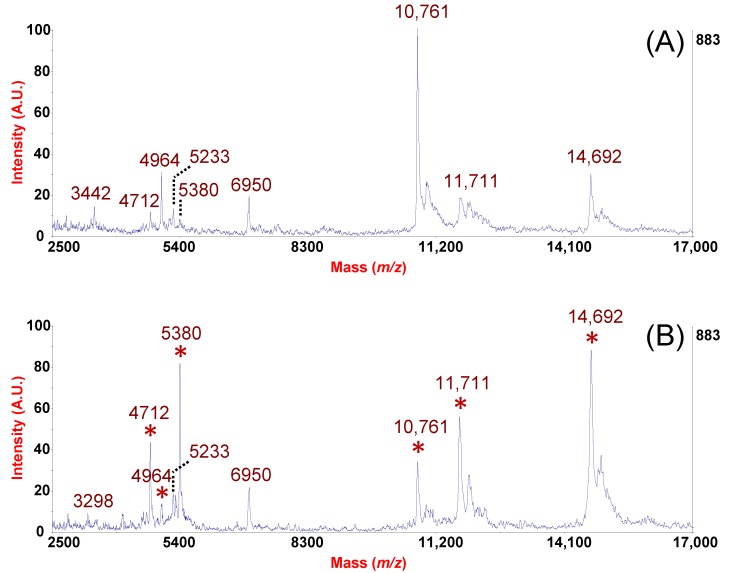
Rhino-peptidomic fingerprints of NF processed by MPS-C from a patient suffering from NARMA CRS with UNP before (**A**) and after nasal polypectomy (**B**). Peaks of AMP that showed changes in the levels of expression after the removal of nasal polyps are marked by asterisks in panel B.

Figure 3 illustrates the rhinomic fingerprints before and after the nasal polypectomy of a subject suffering from Non Allergic Rhinitis with Mast cells (NARMA) and Chronic Rhino-Sinusitis (CRS) with Unilateral Nasal Polyposis (UNP). Both profiles, obtained after processing an equal amount of NF with MPS and spotting equal volume of sample on MALDI target plate in the same experimental session, were compared in absolute units for peak intensity. It is interesting to observe the marked increase (more than three-fold) of Lysozyme (*m*/*z* = 14,692) as an index of inflammation post polypectomy. A similar trend was observed also for SLPI (*m*/*z* = 11,711) and for Statherin and its fragments (*m*/*z* = 5380, 5233, 4712, Figure 3). On the contrary, after polypectomy, a decrease in the expression levels was observed for Thymosin-β4 (*m*/*z* = 4964) and β-MSP (*m*/*z* = 10,761), respectively. On the basis of a recent study, which revealed a correlation between fungal activity and the pathogenesis of chronic sinusitis with NP [35], we hypothesize the potential antifungal role of β-MSP in the nasal cavity. The elevated levels of expression of β-MSP before surgical treatment may be explained as an attempt from the cells of nasal mucosa to contrast the fungal infection while the reduction of its levels after Functional Endoscopic Sinus Surgery (FESS) may be explained as a gradual return to the normal functions of the nasal mucosa resulting from the surgical removal of nasal polyps. This aligns with the previously mentioned hypothesis of the antifungal activity of β-MSP in the nasal cavity in patients suffering from CRS with NP. The spectra comparison offers us the possibility to visualize the variations of an interesting repertoire of endogenous polypeptides with antimicrobial, immunity-related, and still not completely understood functions. Among different approaches developed to investigate NP mechanisms, the chance to extend the study on a larger cohorts (NP vs. absence of NP or NP before and after polypectomy) and to statistically analyze any variation of these important mediators by our tool could constitute a viable opportunity.

**Figure 4:**
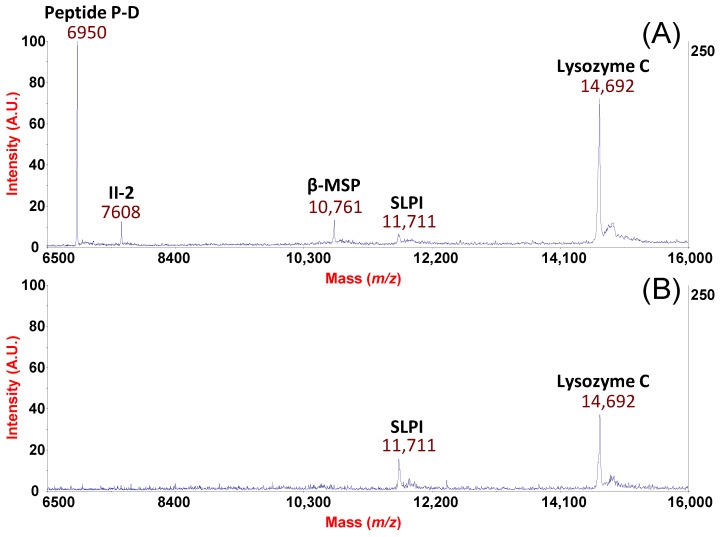
Differential spectra of NF processed by MPS from a patient suffering from AR with no treatment (**A**) and post-treatment (**B**) with azelastine and fluticasone.

In a further example of potential applications of our platform, we also show the case of a patient suffering from AR and is topically treated for 30 days with antihistamines and corticosteroids (azelastine hydrochloride and fluticasone propionate). Figure 4 illustrates the rhinomic fingerprints obtained by our procedure before the pollen season without pharmacological treatment (Figure 4A) and after 30 days of treatment (Figure 4B). The signal loss for peptide P-D and II-2 and a decreased expression of β-MSP and Lysozyme-C are quite evident from spectra comparison (Figure 4 panel A vs. B). It is well known that Lysozyme-C is an index of serous glandular secretions [36]. In our previous work [7], we found a statistically significant increased expression of Lysozyme-C in the NF of AR patients (also in this case out of pollen season) in comparison to healthy subjects. Therefore, it can be speculated that allergic subjects may display hypertrophy or hyper-activation of the nasal submucosal glands and the use of topical corticosteroids suppress cellular infiltrates in the edematous nasal tissues [37] as well as the release of inflammatory mediators [36]. This speculation might also explain the observed decreased level of β-MSP or the lack of AMP peptide P-D and II-2.

**Figure 5:**
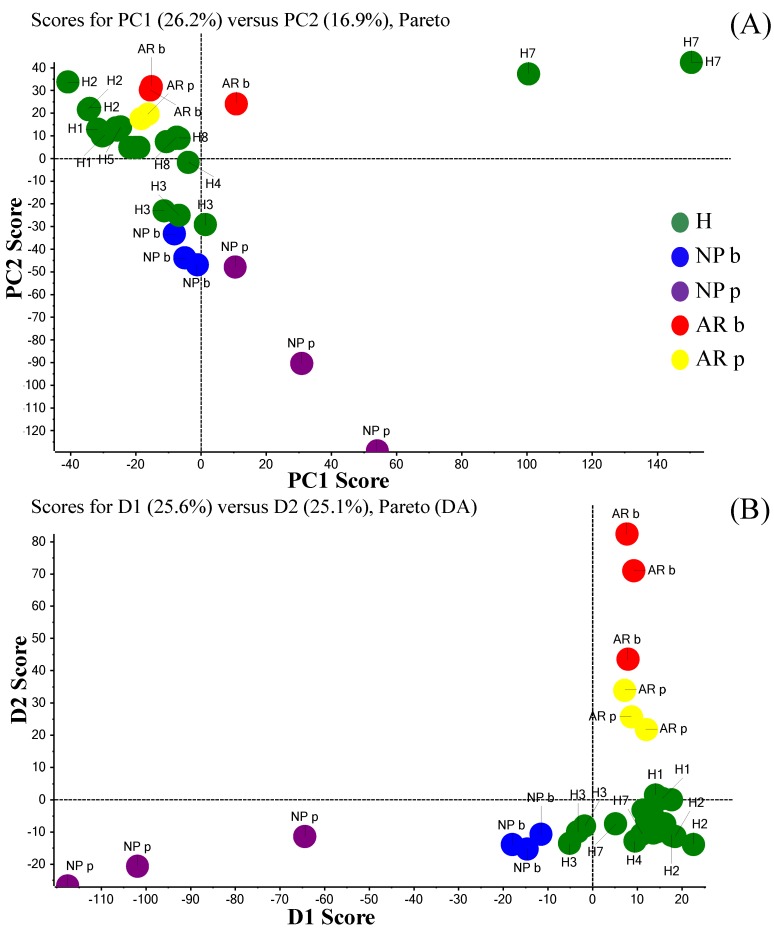
Unsupervised PCA scores plot (panel (**A**)) and the corresponding PCA-DA (supervised) scores plot (panel (**B**)) to visualize NF samples clustering. The green dots represent the NF sample obtained from eight healthy subjects (H). Red and yellow dots represent the patient with AR before and after treatment with azelastine/fluticasone (AR b and AR p respectively). Blue and violet dots represent the NARMA patient with NP before and after polypectomy (NP b and NP p, respectively). The three colored dots for each individual subject category represent the three independent intra-day replicate analyses for NF samples.

It is clear that neither conclusions nor speculations can be hypothesized because the example shown here are solely isolated cases. However, the strength of the data is because the comparison between two states is performed on the same subject. Additionally, in order to improve quantitative analysis of peptides, we plan the use of internal standards and/or ionic liquid matrices in future experiments. It would be possible for future studies on a larger cohort not only to investigate every individual variation post polypectomy or post pharmacological treatment on a longitudinal scale but also to assess if intra-individual differences of AMP patterns might have a statistical significance. Therefore, just to have an exploratory visualization of our preliminary data, we used principal component analysis (PCA) including healthy individuals and the two patients discussed before. An individual subject category is shown by letters (H for healthy, AR for the allergic subject, and NP for the nasal polyposis patient) and colored dots (Figure 5). Despite the previously highlighted inter subject variability (Table 1, Figures S1 and S2), healthy individuals (from H1 to H8) are quite tightly clustered in the unsupervised analysis except for subject H7 likely due to the overexpression of peptide P-D (normalized peak area of 33.62 compared to an average of 6.65, see Figure S2) in this individual. This contributes to the very high variability observed for this molecular species (Figure S2). Therefore, this participant has to be considered as an outlier. The allergic individual after azelastine/fluticasone treatment (yellow dots) is plotted in the healthy zone in the unsupervised PCA (Figure 5A). It appears that the effect of the pharmacological treatment is to re-establish the altered parameter of AMP found in the allergic subject even out of the pollen season to values more similar to those observed in the case of healthy subjects.

The polyposis patient (violet and blue dots) and the allergic subject before treatments (red dots) were more distant in the plot both from each other and from the healthy subjects (green dots), which indicates a higher variability of expression when compared to the healthy individuals. Samples clustering and individual differentiation is better visualized in the supervised analysis, which confirms data homogeneity (Figure 5B) and strongly suggests that, even on a small size group, our platform might offer a useful baseline resource for future AMP biomarker studies. In particular, stratification biomarkers discovery could facilitate the understanding of the individual driving mechanisms of pathology as well as the identification of drug targets potentially useful for tailored treatments of respiratory disorders. However, the translational process consisting of information transfer from basic research to the real-life clinical setting is still in its infancy. Many years will be needed to perform larger investigations able to validate and extend the utilization of proteomic/peptidomic approaches for tailored patient healthcare. Accurate molecular signatures extrapolated from NF collected with improved and standardized procedures could positively affect the understanding of the physio-pathogenesis of nasal inflammation or infection and the underlying mechanisms of immunotherapy as well as potentially open new avenues for diagnostic tools. From a clinical viewpoint, this study highlights the importance of NF as a useful and non-invasive source of biomarkers for monitoring the inflammatory status of subjects suffering from respiratory disorders and for future personalized pharmacological therapies.

## 3. Materials and Methods

### 3.1. Reagents

MPS-C was prepared according a procedure described in Reference [27]. The MALDI matrices SA and alpha-cyano-4-hydroxycinnamic acid (CHCA) were purchased from Fluka (St. Louis, MO, USA). AB SCIEX Peptide Mass Standards Kit for Calibration (AB SCIEX, Framingham, MA, USA), protease inhibitor cocktail (PIC; P8340, Sigma, St. Louis, MI, USA), and Bio-Rad Protein Assay Kit (Bio-Rad Laboratories, Hercules, CA, USA) were used according to the manufacturer’s instructions. The water was obtained from a Milli-Q water purification system (Millipore, Bedford, MA, USA). Acetonitrile (ACN) (HPLC grade) and trifluoroacetic acid (TFA) (ACS grade) were obtained from Merck (Darmstadt, Germany). DTT and sodium chloride were purchased from Sigma-Aldrich (St. Louis, MO, USA). Apart from CHCA that was recrystallized, the other reagents were used as they were received.

### 3.2. Patient Recruitment

All subjects including eight healthy, one suffering from AR, and one suffering from NARMA CRS with UNP were recruited from the operative unit of Otorhinolaryngology of Magna Græcia University and were enrolled in this study after the informed written consent was obtained from each participant, according to the Institutional Ethical Committee Board (Approval number 219, 16 November 2016). Clinical characteristics of the subjects enrolled in this study are listed in Table 2. All subjects underwent an anamnesis, otorhinolaryngological evaluation. The clinical evaluation included a complete traditional ear nose throat examination of the upper airways and an endoscopic examination with a flexible fiber rhinolaringoscope (Olympus Winter & Ibe GmbH, Berlin, Germany) of the nasal and nasopharyngeal cavities. Nasal cytology was also performed for all donors by nasal scraping using a Rhino-Probe R 17 18, according to the method by Gelardi et al. [38]. Healthy donors had negative skin test results to the panel of allergen extracts, did not have allergy histories, and had no history of rhinitis symptoms (congestion, sneezing, itching, rhinorrhea) without NP and infectious rhinopathy.

An AR patient was diagnosed with a skin prick test (Lofarma, Milan, Italy) and a specific IgE (Immulite 2000 XPi Siemens Healthcare, Milan, Italy) measurement. In particular, allergic sensitization was assessed by a skin prick test using a standardized kit of allergens (100 DBU/mL) including *Parietaria officinalis*, *Olea europea*, *Graminacee* mix, *Dermatophagoides* mix, *Compositae* mix, *Cynodon dactylon*, derived epithelial cat and dog, *Cupressus arizonica*, and *Alternaria alternata*. The NF sample was collected before the pollen season (without pharmacological treatment) and after 30 days of azelastine/fluticasone treatment. The patient with left unilateral grade 3 NP with severe respiratory obstruction underwent to FESS in which polypectomy, partial uncinectomy, and antrostomy along with anterior and posterior ethmoidectomy were performed. The NF sample was collected before the patient underwent surgery and two months after surgery, according to the protocol described in Section 3.3.

### 3.3. NF Collection by Nasal Swab

NF was obtained by a non-invasive procedure by cotton swab (Biomèrieux, Marcy-l’Etoile, France) excluding the use of decongestant or topical anesthesia before collection. The device was gently introduced in the nasal fossae covering the middle portion of the inferior turbinate on both sites and then rotated for 5 s. A light pressure was applied on the outside of the nose while rotating the swab to avoid bleeding. The swab containing the NF was immediately placed in a Falcon tube, dipped on ice, and immediately carried to the laboratory for analysis.

### 3.4. NF Recovery from Nasal Swabs

The recovery of NF from the swabs was performed by a three-step centrifugal elution with a hypertonic saline solution (30% sodium chloride), as previously reported [7]. The elution volume was determined as a function of the total amount of the NF collected on the swab (30 μL per mg of the weighted sample). The eluted solutions were then pooled and a Bio-Rad Protein Assay Kit was used to extrapolate protein concentration. The pool was aliquoted and a part was supplemented with PIC in a 1:100 *v*/*v* ratio. All the aliquots with and without PIC were stored at −80 °C except for the samples immediately analyzed by MALDI-TOF MS.

### 3.5. NF Samples Normalization

The concentrations of eluates from nasal swabs from each donor subject ranged from 0.8 to 3.5 μg/μL. A total amount of 75 μg of unfractionated protein from each clinical sample was either concentrated (by vacuum centrifugation) or diluted (by the addition of deionized water) to 30 μL in order to obtain a final concentration of 2.5 μg/μL for further processing with MPS described below.

### 3.6. NF Processing by MPS-C

MPS-C. 2 mg of aminopropyl functionalized MPS-C, preconditioned with 50 μL of 25 mM Tris HCl at pH 8.8 for 5 min, were mixed with 30 μL of NF eluate and shaken at room temperature for 1 h. The slurry was centrifuged at 2000× *g* for 2 min. The particles were then separated from the supernatant and washed with water (2 × 30 μL). After the last wash, the captured species were extracted into 20 μL of a 1:1(*v*/*v*) mixture of ACN and ammonium formate (100 mM, pH = 3).

### 3.7. MALDI-TOF MS Analysis

The SA MALDI matrix was prepared by dissolving 4 mg in 1 mL of a solution prepared with 50% of ACN in 0.1% TFA. The solution was then sonicated for 1 min and then centrifuged for 5 min at 2000× *g*. NF samples were prepared by a dry-droplet method. In addition, 1 μL of the solution containing the bound peptides was mixed with 4 μL of SA solution prepared as described above and 1 μL of the obtained mixture was spotted on the MALDI target plate (Opti-TOF 384-Well Insert, ABSciex, Framingham, MA, USA). It was allowed to dry at room temperature before insertion into the mass spectrometer. MALDI-TOF mass spectra were recorded on AB SCIEX MALDI-TOF/TOF 5800 mass spectrometer (ABSciex, Framingham, MA, USA). For MALDI MS measurements in SA, the following settings were applied: bin size was set at 4 ns, final detector voltage was 2.070 kV with multiplier value at 0.75, and 2000 laser shots were accumulated for each spectrum. External mass calibration was performed using the 5800 Mass Standards kit (AB SCIEX, Framingham, MA, USA) containing insulin bovine (MH^+^ 5734.59), thioredoxin (MH^+^ 11,674.48), and horse apomyoglobin (MH^+^ 16,952.56). Data Explorer version 4.11 software (AB SCIEX, Framingham, MA, USA) was used for data acquisition and data processing.

### 3.8. Identification of NF AMP 

The peaks of interest were analyzed by MALDI-TOF/TOF MS for sequence identification directly from NF processed with the MPS or alternatively after digestion of selected band from 1D SDS-PAGE of NF proteins.

#### 3.8.1. MALDI-TOF/TOF AMP Sequencing in MPS Processed NF

Thymosin β4, statherin Des^39–43^, and Peptide P-D were directly sequenced by MALDI-TOF/TOF MS. In order to acquire sequence information, the MS/MS measurements were performed based on the following information. The CHCA MALDI matrix was prepared by dissolving 4 mg in 1 mL of a solution prepared with 50% of ACN in 0.1% TFA. The solution was then sonicated for 1 min and centrifuged for 5 min at 2000× *g*. Samples were prepared by the dry droplet method. Additionally, 1 μL of solution containing the NF peptides mixture extracted from MPS-C was mixed with 4 μL of CHCA matrix solution prepared as described above and then 1 μL of the resulting solution was spotted on the MALDI target plate and allowed to dry at room temperature before being inserted into the mass spectrometer. The voltages were set at 8.0 kV and 15.0 kV for the ion source 1 and source 2, respectively. Air was used as the collision gas and MS/MS spectra were acquired at a laser energy setting of 5000–6000.

For Sthaterin Des^39–43^, the experimental mass value of the peptide derived from unspecific cleavage of statherin was compared with average theoretical mass values using the FindPept program (http://www.expasy.org/tools). The mass tolerance between theoretical and experimental mass value was set at 100 ppm. Then the experimental CID-MS/MS ion spectrum was compared to the theoretical MS/MS spectrum generated from the Protein Prospector (http://prospector.ucsf.edu/). The fragment ion tolerance was set at 0.25 Da. More structural details with assigned fragmentation and the loss of the two phosphoric acid groups from the precursor ion are shown in Figure S3.

For Thymosin β4 and Peptide P-D, the MASCOT v.2.5.1 search engine (www.matrixscience.com) was used to compare the TOF/TOF spectra against the Homo sapiens primary sequence database NCBI to determine peptide sequence identities. The search parameters included MS tolerance for precursor ions was set at 150 ppm and 0.8 Da for MS/MS ions. 

#### 3.8.2. HNPs Identification

A total of 10 μL of NF aliquots processed with MPS-C were prepared in reduced conditions with the addition of DTT and then analyzed by MALDI-TOF MS in order to reveal disulfide linkages in this triplet of peptides, according to the procedure reported by Ngo et al. [39] (Figure S4). DTT was added to the NF samples to a final DTT concentration of 10 mM. Samples were heated at 100 °C for 5 min, cooled to room temperature, and centrifuged at 14,000× *g* for 5 min. Then 1 μL of this sample was mixed with 4 μL of SA matrix solution prepared as described above and 0.8 μL of the obtained solution was spotted on the MALDI target plate and allowed to dry at room temperature before MALDI-TOF MS analysis. The samples were analyzed in a linear mode. Furthermore, we performed 1D-SDS PAGE and trypsin-digestion of the band of interest following the procedure described below to unambiguously identify these peptides.

#### 3.8.3. NF Proteins Identification by 1D Gel Electrophoresis and MALDI TOF MS

HNPs, β-MSP, SLPI, and lysozyme were identified by 1D SDS-PAGE and peptide mass fingerprinting. The NF sample was heated for 2 min at 85 °C under reducing conditions, vortex-mixed, and applied to Novex 16% polyacrylamide Tricine Gel (Invitrogen, Carlsbad, CA, USA) on an Xcell SureLock Mini-Cell (Invitrogen). Precision Plus Protein Dual Color Standards (BioRad Laboratories Hercules, CA, USA) and color marker Ultra-low Range (Sigma-Aldrich, St. Louis, MO, USA) were loaded in the molecular weight marker lane. The gel was stained with Coomassie Blue Brilliant G-250 and protein bands of interest were cut out from the whole gel and stored into Eppendorf tubes at 4 °C prior to *in-gel* digestion. Excised bands were de-stained and dehydrated. After reduction and alkylation, the bands were digested by modified trypsin (13 ng/μL in 40 mM ammonium bicarbonate) overnight at 37 °C. Digest solutions were acidified with 5 µL of 5% TFA. Peptides from the gel pieces were sequentially extracted three times in 100 μL of 60% (*v*/*v*) ACN, 0.1% (*v*/*v*) TFA. The supernatants were combined and dried using vacuum centrifugation. Dried peptides were then re-suspended in 5 μL of 50% (*v*/*v*) ACN, 0.1% TFA for the MALDI-TOF MS analysis. Then 1 μL of the re-suspended digested peptide mixture was mixed with 4 μL of CHCA matrix solution prepared as described above and 0.8 μL of the obtained solution was spotted on the MALDI target plate and allowed to dry at room temperature before MALDI-TOF MS analysis. The digested peptide mixtures were analyzed in the reflector mode. For MALDI MS measurements in CHCA, the following settings were applied: bin size was set at 1 ns, final detector voltage was 1.980 kV with a multiplier value at 0.66, and 2000 laser shots were accumulated for each spectrum. External mass calibration was performed by using the 5800 Mass Standards kit (AB SCIEX, Framingham, MA, USA) containing des-Arg1-Bradykinin (MH^+^ 904.4681), Angiotensin I (MH^+^ 1296.6853), Glu-Fibrinopeptide B (MH^+^ 1570.6774), ACTH (clip 1–17) (MH^+^ 2093.0867), ACTH (clip 18–39) (MH^+^ 2465.1989), and ACTH (clip 7–38) (MH^+^ 3657.9294). Protein identification was based on PMF and MS/MS analysis. PMF was performed by comparing the experimentally determined peptide masses against the Swiss-Prot sequence database using the Mascot version 2.5.1 (http://www.matrixscience.com/). Database search parameters included up to one missed tryptic cleavage, cysteine carbamidomethylation as fixed modification, and methionine oxidation as a variable modification. Candidates with Mascot scores greater than the 95% confidence threshold (protein score of 65) were accepted. Protein Prospector (http://prospector.ucsf.edu/) was also used to acquire theoretical masses expected for the digested protein. In the MALDI-TOF/TOF experimental session, the voltages were set at 8.0 kV and 15.0 kV for the ion source 1 and source 2, respectively. Air was used as the collision gas and MS/MS spectra were acquired at a laser energy setting of 4000–5000. MS/MS data were calibrated against the MS/MS fragments of the *m*/*z* 1570.677 Glu-Fibrinopeptide B in the standards. The obtained MS/MS data were searched for Homo Sapiens origin against Swiss-Prot database using the MASCOT v.2.5.1 search engine (www.matrixscience.com) with the following parameters: cysteine carbamidomethylation and methionine oxidation as fixed and variable modifications respectively, MS tolerance for precursor ions, 50 ppm, and MS tolerance for fragment ions,0.25 Da.

### 3.9. PCA

Unsupervised and supervised PCA was performed with the use of MarkerView™ software 1.2.1.1 (AB Sciex, Foster City, CA, USA) in order to visualize samples clustering. Pareto scaling and no weighting was applied on the MALDI-MS data set. The spectral data were processed using the following parameters: mass tolerance 100 ppm, minimum required response 10.0, and maximum number of peaks 5000. 

## Figures and Tables

**Table 1:** NF proteins identified using MALDI-TOF/TOF MS.

Swiss Prot Number	Protein Name	Sequence	PI ^d^	Identification Method	Mascot Score ^f^	Peptide Peak Frequency ^g^
P59665/6	HNPs	R.YGTCIYQGR.L ^a^	8.67	NF samples reduction with DTT ^e^; 1DE-MALDI-TOF/TOF	68	5/8 (HNP-1) 5/8 (HNP-2) 3/8 (HNP-3)
P02808	Statherin (di-phosph.) Des^39–43^	DSSEEKFLRRIGRFGYGYGPYQPVPEQPLYPQPYQPQY + Phospho (S) ^b^	4.41	MALDI–TOF/TOF	*De Novo* Sequencing	8/8
P63313	Thymosin β10	ADKPDMGEIASFDKAKLKKTETQEKNTLPTKETIEQEKRSEIS + Acetyl (N-Term) ^c^	5.32	MALDI–TOF/TOF	97	4/8
P62328	Thymosin β4	SDKPDMAEIEKFDKSKLKKTETQEKNPLPSKETIEQEKQAGES + Acetyl (N-Term) ^c^	5.02	MALDI–TOF/TOF	100	8/8
P10163	Peptide P-D (P_32_→A)	SPPGKPQGPPQQEGNKPQGPPPPGKPQGPPPAGGNPQQPQAPPAGKPQGPPPPPQGGRPPRPAQGQQPPQ ^c^	11.17	MALDI–TOF/TOF	96	5/8
P10163	Peptide P-D	SPPGKPQGPPQQEGNKPQGPPPPGKPQGPPPPGGNPQQPQAPPAGKPQGPPPPPQGGRPPRPAQGQQPPQ ^c^	11.17	MALDI–TOF/TOF	89	8/8
P04280	II-2	QNLNEDVSQEESPSLIAGNPQGPSPQGGNKPQGPPPPPGKPQGPPPQGGNKPQGPPPPGKPQGPPPQGDKSRSPR + Pyro-glu ^c^	9.40	MALDI–TOF/TOF	100	5/8
P04280	II-2	QNLNEDVSQEESPSLIAGNPQGPSPQGGNKPQGPPPPPGKPQGPPPQGGNKPQGPPPPGKPQGPPPQGDKSRSPR + Phospho (S) + Pyro-glu ^c^	7.35	MALDI–TOF/TOF	100	7/8
P08118	β-MSP	SCYFIPNEGVPGDSTR.K ^a^	5.36	1DE-MALDI-TOF/TOF	76	7/8
P03973	SLPI	R.YKKPECQSDWQCPGK.K ^a^	9.11	1DE-MALDI-TOF/TOF	68	8/8
P61626	Lysozyme C	R.STDYGIFQINSR.Y ^a^	9.28	1DE-MALDI-TOF/TOF	68	8/8

^a^ Protein identification was based on peptide mass fingerprinting (PMF) and MS/MS analysis. Searches were performed under the following parameters for PMF: Taxonomy, H. Sapiens, Mass Tolerance, 50 ppm, Missing cleavages, ≤2; Enzyme, Trypsin, Fixed modifications, Carbamidomethylation, Variable modifications, Oxidation (M), Charge State, 1^+^. Search parameters for MS/MS: MS Tolerance, 50 ppm. MS/MS tolerance, 0.25 Da. Enzyme, Trypsin. Charge state, 1^+^. ^b^ Statherin fragment identification was based on MS/MS analysis and *De Novo* sequencing was performed to assign amino acid sequences. ^c^ Proteins identification was performed directly by MS/MS experiments. Searches were performed under the following parameters: MS Tolerance, 150 ppm, MS/MS tolerance, 0.8 Da, Enzyme, None, Charge state, 1^+^. ^d^ To calculate the Isoelectric Point we used the prediction tool: Compute PI/MW from EXPASY (http://web.expasy.org/compute_pi/). To calculate the Isoelectric Point of Phosphorylated Proteins we used the prediction tool: Calculate Molecular Weight and Isoelectric Point from SCANSITE (http://scansite3.mit.edu/#util-calcMwAndPi). ^e^ Dithiothreitol (DTT) was added to NF samples to reveal disulfide bonds of HNPs as reported in Supplementary Material. ^f^ Ions score in an MS/MS ion search (significance threshold *p* < 0.05). ^g^ Number of subjects showing the presence of the analyzed peptide.

**Table 2:** Clinical characteristics of the examined subjects.

Subject	Gender	Age	Nasal Cytology ^a^
Healthy 1	F	19	Negative
Healthy 2	F	23	Negative
Healthy 3	M	38	Negative
Healthy 4	F	22	Negative
Healthy 5	F	39	Negative
Healthy 6	F	28	Negative
Healthy 7	F	30	Negative
Healthy 8	F	19	Negative
AR	M	22	M+
NARMA CRS UNP	M	60	M+++

^a^ M, mast cells infiltration.

## Supplementary Materials

The Supplementary Materials are available online at http://www.mdpi.com/1422-0067/19/12/4005/s1.
