# Supplementary material for: Rapid Detection and Identification of Antimicrobial Peptide Fingerprints of Nasal Fluid by Mesoporous Silica Particles and MALDI-TOF/TOF Mass Spectrometry: From the Analytical Approach to the Diagnostic Applicability in Precision Medicine
Source: Int J Mol Sci. 2018 Dec 12;19(12):4005. doi: 10.3390/ijms19124005 (PMC6320778; doi:10.3390/ijms19124005)
Supplement: Supplementary file 1 [file ijms-19-04005-s001.pdf]

Supplementary Materials

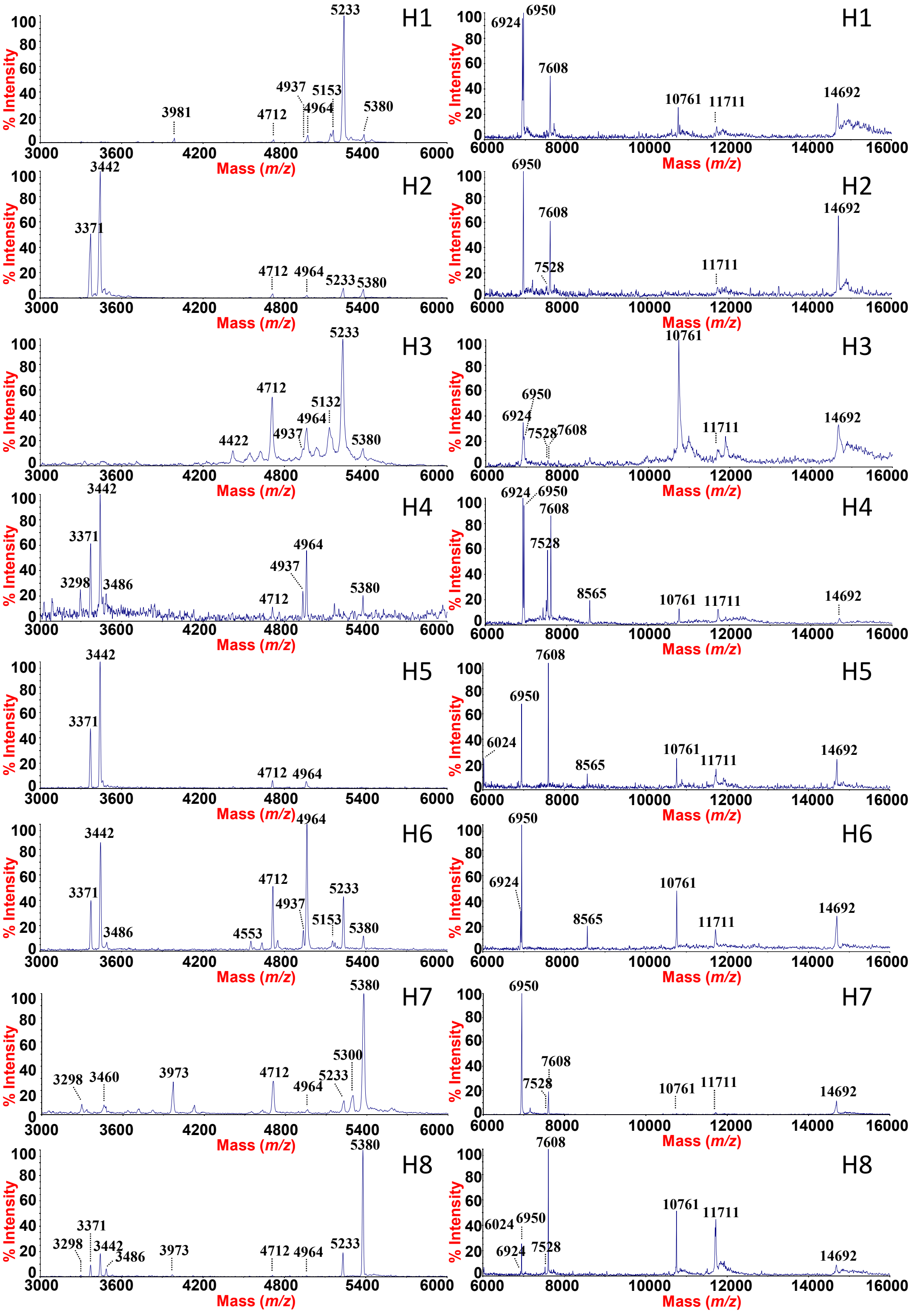

Figure S1. MALDI-TOF fingerprints comparison of NF profiles obtained from eight healthy subjects. MALDI measurements were performed in SA and the spectra were recorded in linear mode. *m/z* ranges of MALDI-TOF spectra from 3000 to 6000 (left panels) and from 6000 to 16000 (right panels) are compared for best detection of molecular features.

Table S1 Reproducibility assessment for normalized area, height and S/N of selected peaks in acquired MALDI-TOF MS spectra from replicate analyses (n=3) of NF specimens processed by MPS.

	MPS-C		
m/z	CV[a)] % Normalized Area	CV % S/N	CV % Height
3371	5	11	8
3442	6	10	8
3486	8	10	13
4712	7	11	13
4937	8	8	14
4964	10	9	11
6924	14	13	11
6950	11	13	11
7528	16	12	12
7608	11	9	8
10761	11	11	15
11711	10	13	13
14692	12	17	13

a) The mean percentage CV was calculated from the ratio between SD and the mean of peak height, peak normalized area, and S/N.

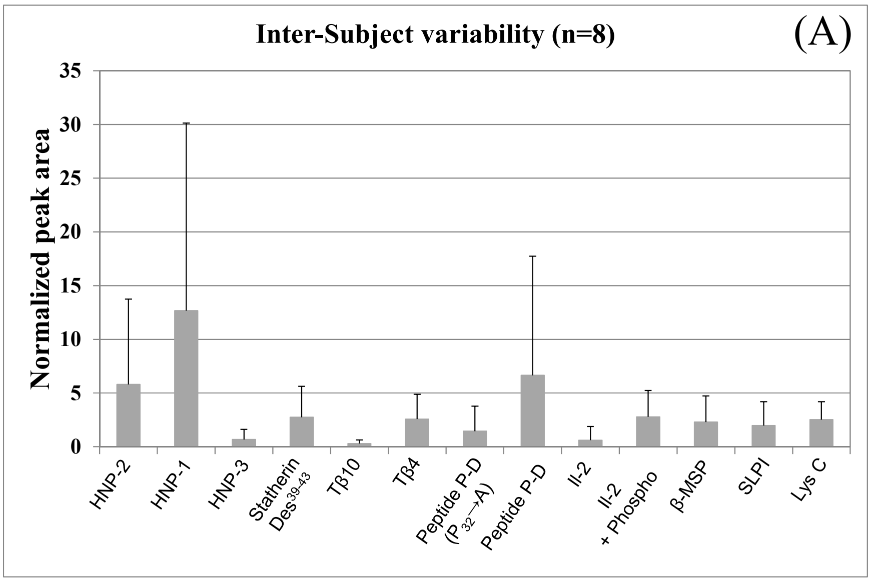

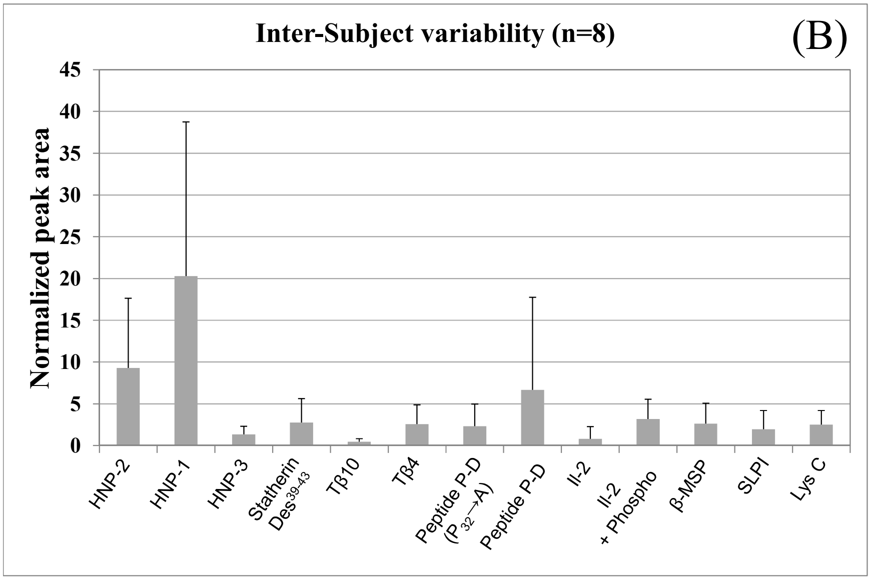

Figure S2. Normalized peak area values with the correspondent standard deviation was plotted for the main peaks enriched by MPS-C, to reveal inter-subject variability in the case of healthy subjects (n=8). In panel B, the normalized peak area was assessed excluding from the mean calculation those subjects which did not show the presence of the analyzed peak.

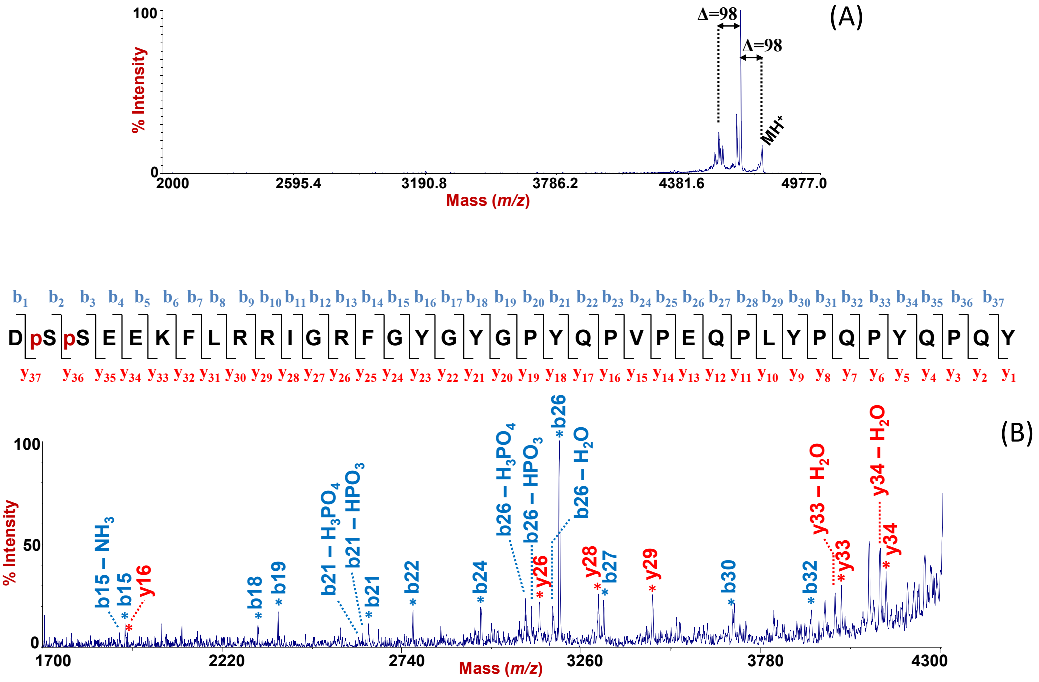

Figure S3 MALDI-TOF/TOF mass spectra obtained by direct sequencing of statherin Des$^{39-43}$ (di-phosphorylated on Ser 2 and Ser 3) from NF sample processed with the MPS. Panel A shows the elimination of phosphoric acid from the precursor ion as the preferred fragmentation pathway (loss of H_3PO_4, Δ=98 Da), which is diagnostic for determining the presence of phosphoserine-containing peptides. In particular, two phosphoric acid groups were lost from the precursor ion, indicating the presence of two phosphorylation sites (serine residues in position 2and 3). Panel B shows the MS/MS spectrum of the phosphorylated peptide that was summed up from 18000 shots to obtain a higher yield of backbone fragment ions. The resulting b- and y-ion series (blue and red respectively), provide complementary sequence information allowing the unambiguous identification of the statherin fragment. Ions b15-b32 series comprise both the intact phosphorylated serine residues at positions 2 and 3 in the peptide. An in-depth analysis of the tandem mass spectrum revealed also the presence of additional fragment ions, such as (b-NH_3), (b-H_2O), (b-HPO_3) and (b-H_3PO_4) series.

HNPs identification

A first attempt to characterize HNPs was performed on the bases of structural features. HNPs are known to contain three intramolecular disulfide bonds [1]. Therefore we used a reducing agent as DTT to reveal disulfide bonds by MALDI-TOF MS. NF samples were prepared in reduced conditions with the addition of DTT and then analyzed by MALDI-TOF MS. As a result of reduction of three internal disulfide bonds, Figure S3 shows a +6 Daltons mass shift of peaks m/z = 3371, m/z =3442 and m/z =3486 after treatment with DTT, suggesting that this well-defined triplet of peptides belongs to the family of human α-defensins. To unambiguously identify these peptides as HNPs we performed 1D-SDS PAGE and trypsin-digestion of band of interest. Trypsin-digested sample yielded a 1117.51 Da peptide, which was further sequenced by MALDI-TOF/TOF MS (Figure 2A) and identified as a conserved sequence among the three HNPs (YGTCIYQGR aminoacid positions 16 to 24).

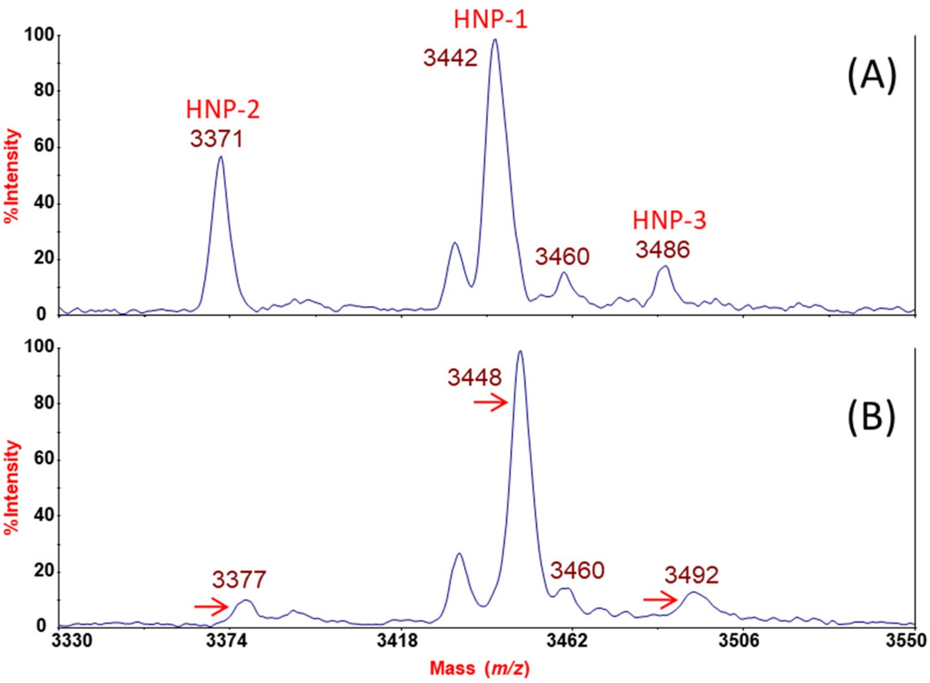

Figure S4 MALDI-TOF mass spectra comparison of the same NF sample before (A) and after (B) DTT addition. The shift in the molecular mass (+6 Da) after treatment with DTT (B) of peaks with m/z 3371, m/z 3442 and m/z 3486, revealed the reduction of three internal disulfide bonds, suggesting that these peptides belong to the family of human α-defensins.

Reference

1. Pardi, A.; Zhang, X.L.; Selsted, M.E.; Skalicky, J.J.; Yip, P.F. NMR studies of defensin antimicrobial peptides. 2. Three-dimensional structures of rabbit NP-2 and human HNP-1. *Biochemistry* **1992,** *31*, 11357-11364. [PubMed]
